# In Vitro and In Vivo Evaluation of the De Novo Designed Antimicrobial Peptide P6.2 Against a KPC-Producing *P. aeruginosa* Clinical Isolate

**DOI:** 10.3390/biom15030339

**Published:** 2025-02-27

**Authors:** Melina M. B. Martinez, Merlina Corleto, Melanie Weschenfeller, Santiago Urrea Montes, Camila N. Salomón, Natalia Gonzalez, Matías Garavaglia, Diego Faccone, Paulo C. Maffía

**Affiliations:** 1 Laboratorio de Aplicaciones Biotecnológicas y Microbiología (LAByM), Secretaría de Investigación, Universidad Nacional de Hurlingham (UNAHUR), Hurlingham 1688, Buenos Aires, Argentina; melina.martinez@unahur.edu.ar (M.M.B.M.); merlina.corleto@unahur.edu.ar (M.C.); melanie.weschenfeller@unahur.edu.ar (M.W.); santiago.urreamontes@unahur.edu.ar (S.U.M.); camilanaiarasalomon@gmail.com (C.N.S.); gonzalezn35395@gmail.com (N.G.); matias.garavaglia@unahur.edu.ar (M.G.); 2 Consejo Nacional de Investigaciones Científicas y Técnicas (CONICET), Ciudad Autónoma de Buenos Aires 1425, Argentina; dfaccone@anlis.gob.ar; 3 Comisión de Investigaciones Científicas de la Provincia de Buenos Aires (CIC), La Plata 1900, Buenos Aires, Argentina; 4 Servicio Antimicrobianos, National Reference Laboratory in Antimicrobial Resistance (NRLAR), National Institute of Infectious Diseases (INEI), ANLIS “Dr. Carlos G. Malbrán”, Ave. Velez Sarsfield, 563, Buenos Aires City 1281, Argentina

**Keywords:** antimicrobial peptides, *Pseudomonas aeruginosa*, meropenem, KPC

## Abstract

The antimicrobial peptide P6.2 was previously de novo designed as an alpha helix cationic amphipathic molecule. In previous work, we have shown that this peptide displayed significant antimicrobial activity against both Gram-positive (*Staphylococcus aureus*) and Gram-negative (*Pseudomonas aeruginosa*) bacteria. However, while P6.2 lacked biofilm-inhibiting properties against the *P. aeruginosa* strain PA01, it displayed anti-inflammatory effects in a murine acute lung infection model challenged with this pathogen. In this work, the peptide P6.2 antimicrobial activity and its possible synergy with meropenem were evaluated both in vitro and in vivo using a *Galleria mellonella* infection model against a carbapenem-resistant KPC-producing clinical isolate of *P. aeruginosa*. Firstly, the cytotoxic effect of the peptide on A549 and RAW264.7 cell lines was assayed, showing no cytotoxicity at 64 µg/mL and below. Then, the MIC (minimal inhibitory concentration) and bactericidal effect against the carbapenemase-producing strain *P. aeruginosa* M13513 strain were determined. P6.2 showed a MIC between 32 and 64 µg/mL, and a rapid bactericidal activity against this strain (less than 45 min). The peptide stability at different temperatures and in bovine serum at 37 °C was also analyzed, showing good stability and almost no degradation after 15 min of incubation at 100 °C or 24 h at 37 °C in serum, respectively. The antibiofilm activity was also evaluated, and although the peptide did not show biofilm inhibitory activity, it did demonstrate biofilm disruptive activity, together with bactericidal activity inside the pre-formed biofilm. The possible synergistic effect with the carbapenem meropenem was then analyzed in vitro by killing kinetics, revealing a synergistic interaction between P6.2 and the antibiotic against this strain. Finally, P6.2 was evaluated in vivo in the *Galleria mellonella* larvae infection model. Interestingly, in *G. mellonella,* P6.2 alone did not completely clear the infection caused by *P. aeruginosa* M13513. However, when combined with meropenem, P6.2 demonstrated a synergistic effect, leading to increased survival rates in infected larvae. The results presented here highlight the potential that this peptide displays when used in combination with carbapenems against a clinically relevant KPC-producing *P. aeruginosa*.

## 1. Introduction

In the last few years, the decreasing effectiveness of conventional antimicrobial-drugs has caused serious problems due to the rapid emergence of multidrug-resistant pathogens [1,2]. This situation has brought attention to other antimicrobial agents, like antimicrobial peptides (AMPs), as they are considered an alternative to conventional drugs [3,4]. These compounds target bacterial membranes for their activity, which gives them a broad spectrum of action and less probable resistance development [5,6,7]. Also, this mechanism of action elicits faster bactericidal activity compared to conventional antibiotics [8]. Furthermore, in the context of the latest pandemic threats, it is imperative to develop new strategies to offer effective antimicrobial therapies for patients with long-term hospitalization [9,10].

Carbapenem-resistant *Pseudomonas aeruginosa* is classified as a high-priority group on the WHO Bacterial Priority Pathogens List, due to its global threat [11,12]. This list established a guide of pathogens to invest in the research and development of treatments, but also strategies to prevent and control antimicrobial resistance in these pathogens. KPC is a worldwide-spread plasmid-borne carbapenemase which confers resistance to all beta-lactams, including carbapenems [13].

Peptide 6.2 (P6.2) is a de novo designed antimicrobial peptide, with an amphipathic alfa helical structure. In previous work, we have demonstrated the antimicrobial and antibiofilm activity of P6.2 against Gram-positive and Gram-negative bacteria [14]. Also, we showed that this AMP has low hemolytic activity, reaching less than 10% of human and mice erythrocytes lysis, within the minimal inhibitory concentration (MIC) ranges [15]. We also tested the affinity towards eukaryotic and prokaryotic membranes showing selectivity for bacterial membranes in agreement with the experimental results [14].

In this work, we have tested the antimicrobial and anti-biofilm activity of P6.2 against KPC-producing *P. aeruginosa* M13513, a carbapenem-resistant strain, alone or in combination with meropenem, in vitro and in vivo in the *Galleria mellonella* model.

## 2. Material and Methods

### 2.1. Peptide Synthesis

The peptide was synthesized with C-terminal amidation and purified to a grade of >95% using HPLC (GenScript Co., Piscataway, NJ, USA).

The sequence of the peptide, together with a helical wheel projection, 3D structure, and physicochemical parameters, are disclosed in the Supplementary Materials.

### 2.2. Bacterial Strain and Growth Conditions

*Pseudomonas aeruginosa* M13513 is a clinical isolate carrying the carbapenemase KPC-2 (*blaKPC-2* gene). This strain was generously supplied by the Servicio Antimicrobianos, Instituto Nacional de Enfermedades Infecciosas (INEI)-ANLIS “Dr. Carlos G. Malbrán” in Buenos Aires, Argentina [16].

For antimicrobial testing, bacterial cultures were maintained in Cation-Adjusted Mueller–Hinton Broth (CAMHB), following Clinical and Laboratory Standards Institute (CLSI) guidelines.

### 2.3. Antimicrobial Activity

The minimal inhibitory concentration was assessed using a standard microdilution assay, following CLSI guidelines [15]. Mueller–Hinton Broth (Laboratorios Britania S.A., Buenos Aires, Argentina) supplemented with Ca^2+^ (20–25 mg/L) and Mg^2+^ (10–12.5 mg/L) was used for the assay. The bacterial strains tested included *Pseudomonas aeruginosa* PAO1, *Pseudomonas aeruginosa* M13513, and *Staphylococcus aureus* ATCC^®^ 25923.

### 2.4. Bactericidal Effect

The bactericidal activity was determined according to the CLSI recommendations [17]. In short, the bactericidal activity of the antimicrobial agents on the microorganisms was evaluated using a time–kill curve. The Minimal Bactericidal Concentration (MBC) is the lowest drug concentration required to eliminate 99.9% of viable organisms (a 3-log reduction in CFU/mL compared to the control) after a set incubation period under defined conditions. This is the most common method for estimating bactericidal activity.

### 2.5. Peptide Stability

To analyze the stability of the molecule, the peptide was exposed to different temperatures for varying durations. The conditions tested involved incubation at 37 °C for 24 h, at 60 °C for 2 h, and at 100 °C for 15 min. Each condition was set up in a microtube with the peptide dissolved in sterile deionized water. As a control, the peptide was dissolved immediately before testing without temperature incubation. Afterward, the broth microdilution assay was conducted to assess its antimicrobial effectiveness. Additionally, the stability of the peptide in serum was examined. Three microtubes were prepared, each containing sterile deionized water with FCBS (fetal calf bovine serum) 25% (*v*/*v*) and the antimicrobial peptide. Two of these samples were incubated at 37 °C for 24 h or 2 h, while the third sample was prepared just before testing without any temperature incubation. As controls, serum alone and peptide without serum were used.

The antimicrobial activity was subsequently evaluated through the broth microdilution assay.

### 2.6. Cytotoxicity

The toxic effect was evaluated indirectly using the colorimetric MTT assay (3-[4,5-dimethylthiazol-2-yl]-2,5-diphenyl tetrazolium bromide, Thermo Fisher Scientific Inc, Massachusetts, USA) on A549 (human lung adenocarcinoma, ATCC CCL-185) and RAW 264.7 (murine macrophage-like ATCC TIB-71) cell lines. Cell lines were kindly provided by Instituto de Virología e Innovaciones Tecnológicas (IVIT), (INTA-CONICET, Buenos Aires, Argentina).

For the assay, A549 cells were seeded in 96-well plates at a density of 1 × 10^4^ cells per well, while RAW 264.7 cells were seeded at a density of 5 × 10^4^ cells per well. The plates were incubated for 24 h at 37 °C with 5% CO^2^ to allow cell adhesion. The peptide P6.2 was dissolved in distilled water at a stock concentration of 10.24 mg/mL, and serial dilutions were made in RPMI-1640 medium supplemented with 10% fetal bovine serum (FBS, Internegocios S.A., Mercedes, Argentina). The concentrations used to assess P6.2 toxicity were the same as those previously employed in bacterial studies to determine the minimum inhibitory concentration (MIC) in antimicrobial activity assays. For the A549 cell line, P6.2 concentrations ranged from 4 µg/mL to 1024 µg/mL using serial dilutions in culture medium. For the RAW 264.7 cells, P6.2 concentrations ranged from 256 µg/mL to 8 µg/mL, with the concentration range adjusted based on preliminary results obtained from the A549 cell line.

Peptides were added and plates were incubated for 24 h at 37 °C in a 5% CO_2_ atmosphere. After incubation, the culture medium was removed, and the cells were washed with PBS (Phosphate Buffered Saline pH 7.2) to remove any remnants of the treatments. Next, 100 µL of MTT solution (0.5 mg/mL in PBS) was added to each well, and the plates were incubated for 0.5 to 3 h at 37 °C. During this period, viable cells reduced MTT, forming visible formazan crystals under a light field microscope (Zeiss Primovent, Oberkochen, Germany), indicating the cell metabolism of the compound.

At the end of the incubation, the supernatant was removed, and 200 µL of DMSO was added to each well to dissolve the formazan crystals. The plates were shaken to homogenize the contents, and absorbance was measured at 570 nm using a microplate reader (RT2100, Rayto Life and Analytical Sciences Co., Ltd, Shenzhen, China). Triplicates were run for each treatment. Cell viability was calculated by setting the absorbance value obtained for untreated control cells as 100% viability.

### 2.7. Killing Kinetics

Time–kill assays were performed to evaluate the effect of different concentrations of P6.2 on *P. aeruginosa* M13513. The peptide was tested at concentrations ranging from 8 to 1024 µg/mL, incubated in CAMHB with an initial bacterial load of 5 × 10^6^ CFU/mL in 96-well polystyrene plates, with a final volume of 100 µL per well. Cultures without peptides acted as positive controls, while CAMHB alone was used as the negative control.

The plates were incubated at 37 °C for 20 h in the Cell Imaging Multi-Mode Reader Cytation™5, with absorbance readings at 600 nm taken every 2.5 h under continuous shaking. Optical density was plotted against time, displaying the mean ± SD of two independent measurements for each time point. IC50 (Half-maximal inhibitory concentration) values were determined by calculating the peptide concentration required to decrease CFU/mL by 50% relative to the untreated control cultures. These values were obtained by identifying the concentration at which the best-fit trend lines crossed the 50% survival threshold. A non-linear regression analysis was conducted using GraphPad Prism 5 software.

### 2.8. Inhibition of Biofilm Formation

The assessment of biofilm inhibition was carried out using 96-well flat-bottom polystyrene plates. Antimicrobial agents were diluted in Mueller–Hinton broth through a two-fold serial dilution and subsequently inoculated with a fresh bacterial culture to obtain a final concentration of 5 × 10^5^ CFU/mL, with a total well volume of 100 µL. The plates were then incubated at 37 °C for 24 h. Bacteria cultured without the peptide served as the control.

To evaluate biofilm formation following peptide exposure, the supernatant was carefully removed, and the wells were washed twice with 100 µL of saline solution to eliminate any non-adherent cells. The remaining biofilm was fixed by adding 100 µL of methanol for 15 min, followed by staining with 100 µL of 1% (*v*/*v*) crystal violet ( Sigma-Aldrich, Massachusetts, USA) for 5 min. After staining, the excess dye was discarded, and the wells were rinsed twice with 200 µL of distilled water before allowing the plate to dry at 37 °C for 30 min. Finally, 100 µL of 33% acetic acid was added to each well, and the samples were homogenized by gentle agitation. The absorbance was then measured at 595 nm using a microplate reader (RT2100, Rayto Life and Analytical Sciences Co., Ltd., Shenzhen, China).

### 2.9. Eradication of Pre-Formed Biofilm and Cell Viability

Biofilms of *P. aeruginosa* M13513 were grown in 96-well flat-bottom polystyrene plates using CAMHB for 24 h. Once the biofilms were established, the supernatant was removed, and 100 µL of CAMHB containing two-fold serial dilutions of antimicrobials were added to each well. The plates were then incubated for an additional 24 h at 37 °C.

For each experiment, two sets of plates were prepared: one for biofilm quantification using the crystal violet (CV) staining method described earlier, and another to assess bacterial cell viability within the biofilm using the MTT assay (3-(4,5-dimethylthiazol-2-yl)-2,5-diphenyltetrazolium bromide). To perform the MTT assay, the medium was carefully removed, and biofilms were washed three times with 200 µL of saline solution. Subsequently, 100 µL of a 0.05% (*w*/*v*) MTT solution was added to each well, and the plates were incubated in the dark at 37 °C for 3 h.

Following incubation, the supernatant was discarded, and the resulting formazan crystals were dissolved in 100 µL of DMSO. The samples were then homogenized by orbital shaking for 10 min. Finally, absorbance was measured at 570 nm using a microplate reader.

### 2.10. Confocal Laser Scanning Microscopy

To analyze biofilms using Confocal Laser Scanning Microscopy (CLSM), biofilms were cultivated in Ibidi µ-dish 35 mm, High Glass Bottom, under the same conditions as the biofilm eradication assays, with a final volume of 300 µL. Following the incubation with P6.2, the biofilms were stained using the BacLight bacterial viability kit (L7007, Molecular Probes, ThermoFisher Scientific, Waltham, MA, USA), which differentiates between live and dead cells.

Images were captured using a Zeiss LSM 710 confocal microscope (Oberkochen, Germany) equipped with a Plan-Apochromat 63X/1.40 oil objective. Excitation was achieved using an argon laser (λexc = 458, 488, and 514 nm, maximum power 25 mW) and a DPSS 561-10 laser (λexc = 561 nm, maximum power 15 mW). For three-dimensional visualization, images of the same sample field were obtained at varying z-values (z-stack), maintaining a consistent distance from the central plane (±20 nm).

The proportion of live and dead bacteria was determined by analyzing three representative images from each biofilm sample using the ImageJ software 1.52v with the FIJI plugin.

### 2.11. Synergy Evaluation

#### 2.11.1. Checkerboard

To determine the potential synergy between P6.2 and meropenem, the checkerboard method was performed according to [18], and the fractional inhibitory concentration index (FICI) of each combination was calculated by the standard formula: [(MICAC)/MICA] + [(MICPC)/MICP] = FICA + FICP = FIC index, where MICA and MICP are the MICs of the antibiotic and peptide determined separately, and MICAC and MICPC are the MICs of the antibiotic and peptide when determined in combination.

A preliminary assay was performed with combinations of the peptide and meropenem. Because of the variability of the method, we performed a modified method where combinations that showed growth inhibition were selected and evaluated by triplicate for each combination, as well as for the evaluation of the antimicrobials alone, at MIC and sub-MIC concentrations. Growth and sterility controls were also tested by triplicate. At the end point of the experiment, microbial growth was determined by optical density measurements at 600 nm and the data were represented in a bar chart. The OD measurements plotted correspond to the combinations that showed antimicrobial activity, as well as the growth control and the sterility control (as the maximum and minimum growth, respectively). In addition, the OD data of the antimicrobials at 0.5× MIC were represented. Combinations with lower antimicrobial concentrations or antimicrobials at a concentration < 0.5× MIC did not show growth inhibition.

#### 2.11.2. Killing Kinetics

The possible synergistic interaction between P6.2 and meropenem was assessed through time–kill kinetic assays. These experiments were conducted in 50 mL tubes with a final volume of 10 mL. A log-phase culture of *P. aeruginosa* M13513 was obtained and subsequently diluted in CAMHB to achieve a final bacterial concentration of 5 × 10^6^ CFU/mL. Both P6.2 and meropenem were tested individually and in combination at 0.5× MIC (minimum inhibitory concentration). Untreated bacterial cultures served as controls.

The samples were incubated at 37 °C with constant shaking for 24 h. Aliquots were collected at 0, 1, 2, 3, 4, 8, and 20 h, serially diluted in saline, and plated in triplicate on LB agar using three drops of 50 µL per dilution. The plates were incubated at 37 °C for 24 h to determine colony-forming unit (CFU) counts.

The results were plotted as Log CFU/mL versus time (h). A synergistic effect was defined as a reduction in at least two logarithmic units in bacterial count when comparing the combination treatment with the most effective single-agent treatment after 24 h. A bactericidal effect was determined when the initial inoculum was reduced by three or more logarithmic units.

### 2.12. In Vivo Studies in Galleria mellonella

#### 2.12.1. GM Larvae Rearing

Lepidoptera were reared in the laboratory in a standardized manner. A breeding colony was established, and the different stages of the insect’s life cycle were documented. Adult insects were housed in a plastic container without access to food, allowing for the collection of eggs. Then, eggs and larvae were placed in containers with fresh food, prepared from autoclaved wheat bran (50%), dry yeast (5%), beeswax (6%), honey (26%), and glycerin (13%). In each growth cycle, a pool of larvae was reserved to progress to pupa and adult generation. The larvae and eggs were kept at room temperature during the warm seasons and in an oven with humidity control at 30 °C during the cold seasons. For each experiment, a group of late-stage caterpillars was set aside and kept in the dark at a temperature of 10–12 °C, ensuring their use within one week of collection.

#### 2.12.2. Safety in GM Larvae

To evaluate possible adverse effects of AMP administration, doses of 10, 50, and 250 mg/kg of peptide were tested. Each group consisted of 10 larvae (n = 10). A graduated Hamilton-type syringe with a maximum capacity of 25 μL and 1 μL intervals was used to inject the larvae. The peptide was delivered in a single dose of 10 μL via the last left proleg. The larvae were then incubated at 37 °C, and their survival was monitored over a period of five days.

#### 2.12.3. GM Larval Infection Model

For the in vivo tests, larvae of approximately 2 cm in length and an average weight of 250 mg were used. This average weight was used to calculate the antimicrobial doses. The bacterial inoculum used in the larval infection models was prepared from an ON culture, which was washed twice with physiological saline solution and adjusted by measuring OD_600nm_. The larvae were infected by injecting 10 μL of the bacterial suspension 10^5^ CFU/mL (10^3^ CFU/larva) into the last left proleg. One hour after infection, the antimicrobial was administered in a single dose by injecting 10 μL into the last right proleg (peptide, control antibiotic, or saline solution as infection control). Additionally, each assay included a group of untouched larvae to verify the viability of the insect batch, as well as a group injected with physiological saline solution in each proleg to assess potential damage. Each group consisted of 10 larvae (n = 10). The larvae were incubated at 37 °C for five days, with daily monitoring of mortality events.

#### 2.12.4. In Vivo Synergy in GM

To evaluate in vivo synergy, the same procedure was followed as in the previously described infection models. A bacterial suspension of 10^5^ CFU/mL (10^3^ CFU/larva) was used as an inoculum of *P. aeruginosa* M13513. As part of the antimicrobial treatment, the larvae received meropenem at a concentration sufficient to ensure survival, a combination of meropenem and the peptide under suboptimal conditions to assess potential synergy, and meropenem alone in suboptimal conditions as a control. The larvae were incubated at 37 °C, and their survival was monitored for five days.

### 2.13. Statistical Analysis

The statistical analysis was conducted using GraphPad Prism 5 software (GraphPad Software Inc., San Diego, CA, USA). When appropriate, a one-way ANOVA was applied, followed by Dunnett’s post-hoc test, with significance set at *p* < 0.05. Results are expressed as the mean ± standard error.

## 3. Results

### 3.1. Cytotoxicity

**Figure 1:**
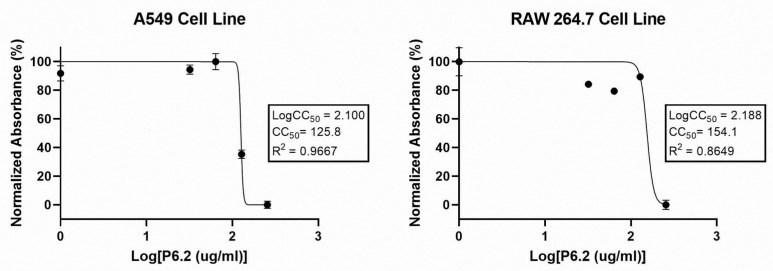
Cytotoxic Activity on A549 and Raw 264.7 cell lines. The graphs show the CC_50_ value (cytotoxic concentration 50%) analyzed by MTT as a percentage calculated considering the untreated cell as 100% of viable cells. Viability was calculated as % of normalized absorbance.

In order to assess the cytotoxic effect on eukaryotic cells, we tested different concentrations (8–256 µg/mL) of P6.2 in vitro against Raw 264.7, a murine macrophage cell line, and A549, the adenocarcinomic human alveolar basal epithelial cell line. The toxic effect of the peptide was evaluated indirectly by the MTT assay on these different cell lines (Figure 1).

In these assays, the CC_50_ value obtained for this peptide in A549 was 125.8 µg/mL, while the CC_50_ in RAW264.7 obtained was 154.1 µg/mL.

The concentration of peptide used for further analysis was established at 64 µg/mL, which is the MIC for *P. aeruginosa* M13513, and also, the concentration between 8 µg/mL and 64 µg/mL, which showed no cytotoxic effects, is the same concentration range observed for the antimicrobial activity of this AMP against different pathogens [19]. These findings are also in accordance with the previous results obtained for toxicity by hemolytic activity (hemolysis below 5% until 128 µg/mL) [15].

### 3.2. Antimicrobial Activity

**Figure 2:**
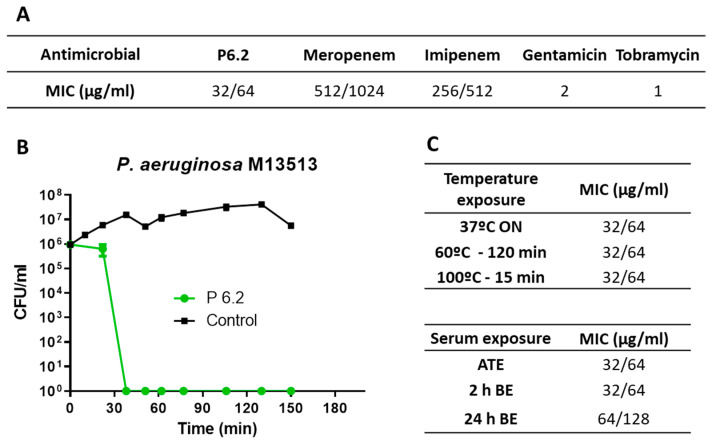
Antimicrobial activity of P6.2 against *P. aeruginosa* M13513. (**A**) MIC determination by broth microdilution assay. Several replicates were performed and two consecutive values are shown for some antimicrobials regarding the acceptable variability of the one-fold dilution method; (**B**) bactericidal effect evaluated by killing kinetics at 64 µg/mL of P6.2; and (**C**) peptide stability at different temperatures and at different incubation times in serum. ATE: P6.2 mixed with serum at the same time as the experiment; BE: P6.2 incubated with serum before the experiment. Control: growth control of *P. aeruginosa* with no antibiotic.

The assessment of antimicrobial efficacy against the *P. aeruginosa* M13513 strain involved the determination of both the minimal inhibitory concentration (MIC) and bactericidal activity. These experiments were performed according to the Clinical and Laboratory Standards Institute (CLSI). P6.2 exhibited a MIC of 64 µg/mL for this resistant strain, comparable to MIC values observed for susceptible strains [14]. Additionally, it demonstrated a remarkably swift bactericidal effect, achieving a 99.9% reduction in viable bacteria (bactericidal definition) within a timeframe of less than 45 min (Figure 2A,B). This is a common effect on the antimicrobial activity of AMPs, which highlights the potential of these molecules as promising new drugs. Gentamicin and tobramycin were used as controls, and the carbapenems were employed to confirm the intrinsic antimicrobial resistance of this KPC-producing strain.

In order to assess the stability of the AMP, P6.2 was incubated at different temperature conditions or with FBS (fetal bovine serum) for different time periods. The temperature exposures were overnight (ON) at 37 °C, 120 min at 60 °C, and 15 min at 100 °C. P6.2 was incubated with 25% FBS at 37 °C for 2 and 24 h before the experiment or directly at the moment of the experiment. Then, a microdilution assay was performed to test the antimicrobial activity. As shown in Figure 2C, only incubation for 24 h with serum seemed to reduce the antimicrobial activity against *P. aeruginosa* M13513, by a two-fold dilution.

### 3.3. Anti-Biofilm Activity

**Figure 3:**
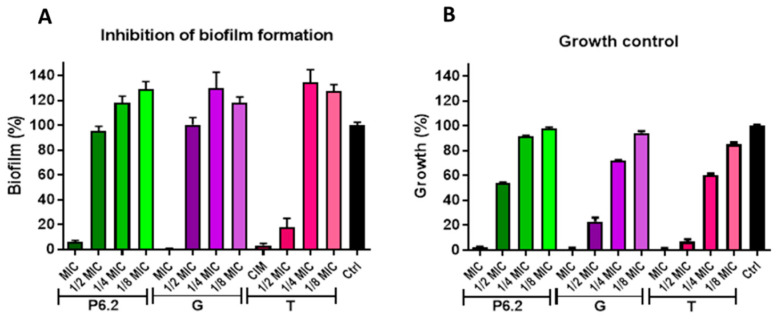
Inhibition of biofilm formation in *P. aeruginosa* M13513. (**A**) Biofilm formation was quantified by crystal violet at different P6.2 concentrations (range 1/8 to ½ of the MIC). (**B**) Bacterial growth was also analyzed under the same treatments as the control. One-way ANOVA with Dunnett’s post-test: comparison of all treatments against the control (C+)G: gentamycin; T: tobramycin.

The biofilm inhibition activity and the pre-formed biofilm disruption activity were evaluated. Figure 3 shows the biofilm inhibitory activity of P6.2 on M13513 strain together with the evaluation of two habitual antibiotics used with *P. aeruginosa*: gentamicin and tobramycin. In these experiments, we could see that the incubation of the peptide with the inoculum at sub-MIC concentrations did not show anti-biofilm activity. We can assume that the decrease in biofilm could be attributed to lower bacterial growth at each peptide concentration.

**Figure 4:**
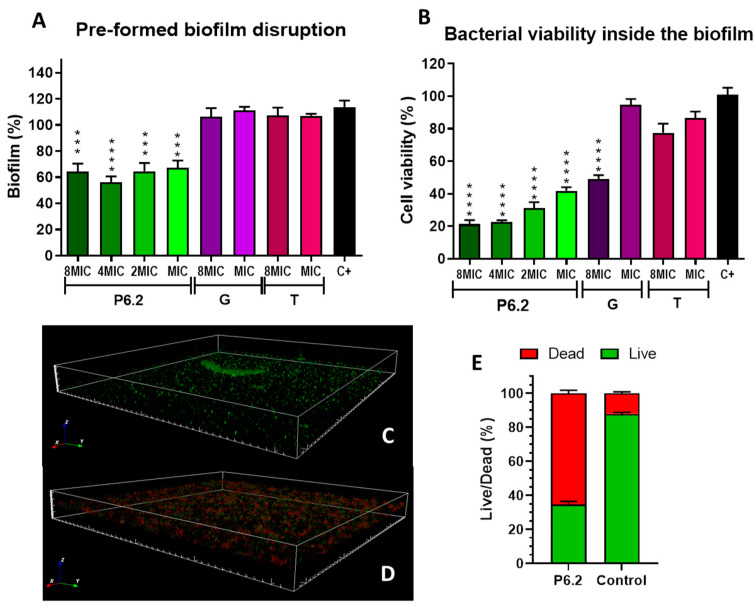
Pre-formed bio film disruption and *P. aeruginosa* M13513 viability. Bacteria were allowed to develop biofilm for 48 h; afterwards, P6.2 or antibiotics were added in a concentration ranging from 1xMIC to 8xMIC. The amount of biofilm was quantified with CV (**A**) and bacteria viability inside the biofilm was analyzed with MTT (**B**). Biofilm analysis by confocal fluorescence microscopy stained with SYTO9 and PI for live (green) and dead (red) cells. The 3D architecture of the control biofilm (**C**) and after P6.2 treatment (**D**) are depicted. The live/dead cells ratio was calculated using ImageJ software (**E**). One-way ANOVA with Dunnett’s multiple comparisons test. Comparison of all treatments against the control (C+); *** *p* < 0.001; **** *p* < 0.0001. T: tobramycin, G: gentamycin.

On the other hand, when tested on a pre-formed biofilm, the peptide not only diminished the biofilm mass, analyzed by crystal violet, but also significantly decreased the bacterial viability inside the biofilm, quantified by MTT assay (Figure 4). These results were also confirmed by confocal fluorescence microscopy, with SYTO9 and PI for live and dead bacterial imaging (Figure 4C–E).

#### 3.3.1. Synergy

**Figure 5:**
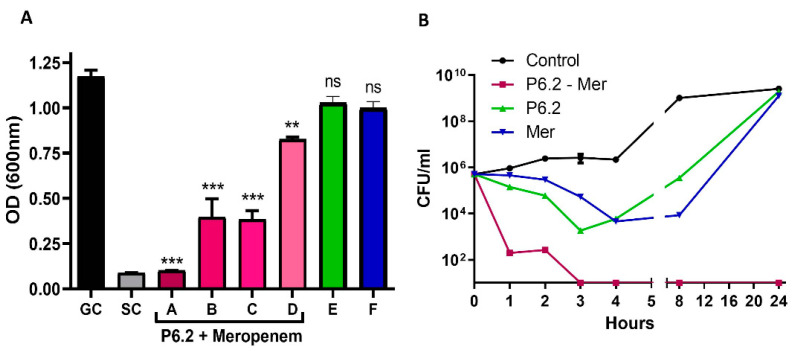
(**A**) Synergistic Activity Using the Checkerboard Method. Bacterial growth was evaluated in each of the combinations of AMPs and meropenem. A: 0.5× MIC P6.2 + 0.5× MIC meropenem; B: 0.25· MIC P6.2 + 0.5× MIC meropenem; C: 0.5× MIC P6.2 + 0.25· MIC meropenem; D: 0.25· MIC P6.2 + 0.25· MIC meropenem; E: P6.2 at 0.5× MIC; F: meropenem at 0.5× MIC; GC: growth control without antimicrobial agents; and SC: sterility control, only CAMHB medium. One-way ANOVA, with Dunnet’s subsequent test, comparing all combinations against the control (C+); *** *p* < 0.001, ** *p* < 0.01. (**B**) Synergistic Activity by Killing Kinetics Method. Each compound and the combination of both was tested at 0.5× MIC. Data were depicted as mean ± SEM of living bacteria vs. time. The data values that were plotted over the “X” axis represent 10^1^ or less CFU/mL (the detection limit of this method).

The potential synergistic activity of P6.2 was additionally assessed in combination with meropenem, in order to ascertain whether this antimicrobial peptide could enhance the antimicrobial efficacy of a drug for which the bacteria exhibit a mechanism of resistance. For this purpose, we first evaluated the potential synergy through an assay based on the checkerboard method [20]. According to the interpretation of FICI, a value of FICI ≤ 0.5 indicates synergy, FICI > 4.0 antagonism, and 0.5 < FICI < 4.0 no interaction. Combination A, which inhibited growth with the absence of turbidity at the naked eye, presented a FICI of 1, which is interpreted as “no interaction”. However, combinations B, C, and D showed slight turbidity (Figure 5A). To be rigorous with the definition, the FICI values could not be calculated in these cases. However, the notable differences in bacterial growth of these combinations compared to the positive control prompted us to hypothesize a possible synergy, due to the evident antimicrobial activity in these cases.

To actually assess the synergy between these two compounds, the killing kinetic method was chosen, which is considered more accurate, reproducible, and better correlated with the in vivo outcomes of antimicrobial agent combinations [21].

In the killing kinetic experiments, the drugs were evaluated at sub-MIC concentrations, alone or in combination (Figure 5B). Synergy is defined as a ≥2-log10 decrease in CFU/mL between the combination and its most active constituent, after 24 h, with the less active component being tested at an ineffective concentration. In addition, the bactericidal effect is considered when the treatment reduces the inoculum in three or more logarithmic units [22]. Figure 5B shows that P6.2 displays synergism with meropenem, both at a 0.5× MIC concentration, and also exhibited a bactericidal effect from 3 h forwards.

#### 3.3.2. In Vivo Evaluation in a *Galleria mellonella* Infection Model

**Figure 6:**
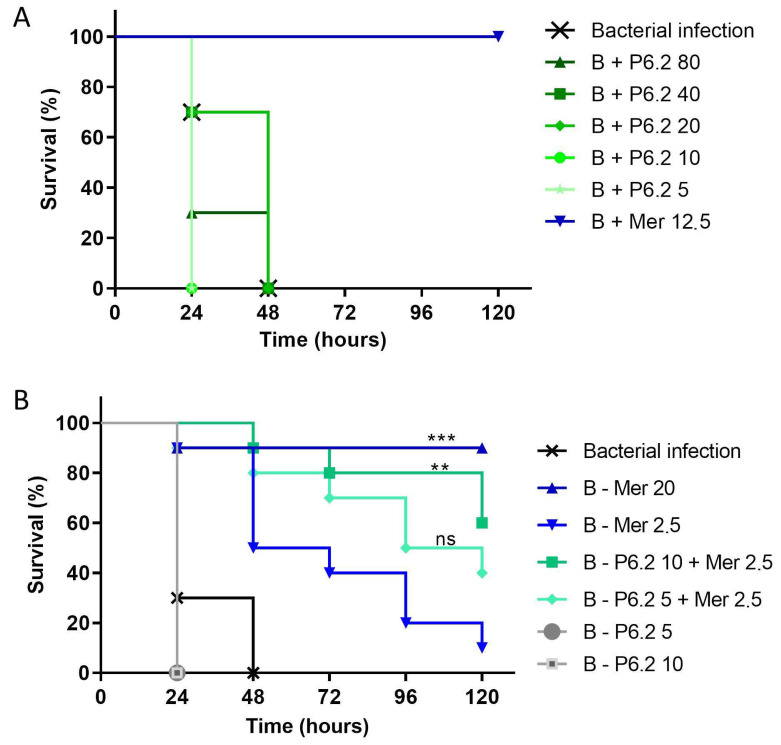
(**A**) In vivo evaluation of P6.2 alone against *P. aeruginosa* M13513. Larvae were infected with 10³ CFU/larva. One hour post infection, P6.2 was injected at 2.5, 10, 20, 40, and 80 mg/kg and meropenem at 12.5 mg/kg. (**B**) In vivo evaluation between P6.2 and meropenem against *P. aeruginosa* M13513. Larvae were infected as above. One hour after infection, peptide P6.2 or antibiotic was administered. B: bacteria; Mer: meropenem. The concentration of each compound is expressed en µg/mL. The data were graphed using the Kaplan–Meier method and comparisons between groups were made using the log rank test. Overall statistical significance was established at *p* < 0.05; and then the Log-rank (Mantel-Cox) test was performed between Mer 2.5 and Mer 2.5 + P6.2 10 ** *p* value = 0.0076, Mer 20 and Mer 2.5 *** *p* value = 0.0007, and Mer 2.5 and P6.2 5 + Mer 2.5, ns: not significative differences.

The antimicrobial activity of P6.2 was evaluated in the larvae infection model *Galleria mellonella.* Larvae were infected with 10^3^ bacteria and, subsequently, the antibiotic meropenem, peptide P6.2, or their combination was injected in the larvae haemolymph. The results showed that the peptide alone (in the range 2.5–80 mg/kg) was not effective in preventing larvae death by *P. aeruginosa* infection (Figure 6A). However, when P6.2 was administered at a concentration of 10 mg/kg together with meropenem at a concentration of 2.5 mg/kg (which is under the effective dose of 20 mg/kg), it indeed kept the survival of the infected larvae at 80% (Figure 6B), showing the suitability of this peptide/antibiotic combination in an in vivo model of infection.

## 4. Discussion

AMPs have garnered significant attention as promising alternatives to conventional antimicrobial agents, particularly in addressing infections linked to antibiotic-resistant bacteria and biofilm-associated pathogens. Consequently, the discovery and design of new AMPs with these capabilities is a critical objective, offering potential advances in overcoming the global challenge of antibiotic resistance [8].

Previous biophysical analyses have demonstrated the lipid selectivity and the bacterial membrane interaction that P6.2 exhibits, also showing a selective affinity for bacterial membranes over eukaryotic ones [14]. The hemolitic activity of the peptide on healthy human red blood cells was previously evaluated [15]. In this work, the cytotoxicity of the peptide was evaluated in two different cell lines, one a human alveolar cell line (A549) and the other a mouse macrophagic cell line (RAW274.6). The first cell line was chosen because *P. aeruginosa* is an opportunistic pathogen that mainly targets individuals with weakened lung defenses, such as patients with cystic fibrosis, bronchiectasis, or advanced chronic obstructive pulmonary disease (COPD). It often causes persistent lung infections, which can exacerbate their condition and become life-threatening due to its capacity to develop resistance to antibiotics. The macrophagic cell line was chosen because this immune cell type is crucial during *P. aeruginosa* chronical infection. Monocyte-derived macrophages are recruited from the bloodstream and differentiate into pro-inflammatory “M1” macrophages at the infection site. In addition, interstitial and alveolar macrophages play a significant role in the macrophage response during *P. aeruginasa* infection [23].

The MTT assay showed that this peptide displayed a moderate to low cytotoxic effect for A549 and for RAW267.4, in good agreement with previous hemolytic assays [15]. The CC_50_ for both cell lines were obtained, with values of 125.8 and 154.1 µg/mL, respectively.

The killing kinetics of an antimicrobial agent allow us to observe how the agent exerts its activity as a function of time, determining viable cells by counting colony-forming units at different time points. Normally, for commonly used antibiotics, this measurement is carried out over a 24-h period, since they reach their maximum activity during this period. However, for AMPs, these times are much shorter; in fact, these antimicrobials exert their maximum activity in just hours or minutes [24]. For this reason, killing kinetics at MIC was carried out by measuring viable bacteria at short intervals over a period of three hours. The killing kinetic curve showed that the peptide displays a rapid bactericidal activity in less than 45 min, in accordance with other active AMPs [25,26].

In the last few years, there has been huge progress in the design and optimization of AMPs; however, peptide stability has emerged as a significant obstacle to the application of these molecules [27]. Peptides that have proved antimicrobial activity in vitro sometimes cannot display proper in vivo activity or can only be applied topically. Systemic administration is challenged by potential peptide degradation due to the proteases present in serum [28]. In this work, we evaluated peptide stability against high temperature and enzymatic proteolysis. The results showed that P6.2 remains active in most of the conditions evaluated, with a slight degradation of its activity only when incubated in serum for 24 h.

Biofilms are complex bacterial communities characterized by extensive intercellular interactions. Biofilm-associated bacteria, including *P. aeruginosa*, are commonly implicated in chronic infections that can persist for years [29]. As such, managing biofilm-associated infections has become a critical focus in antimicrobial therapy, given their resistance to conventional antibiotic concentrations [30]. In this context, certain AMPs have demonstrated the capacity to prevent biofilm formation or eradicate established biofilms; in some instances, additional mechanisms underlying these antibiofilm effects have also been proposed [31]. Particularly, the carbapenem-resistant clinical strain M13513, which was isolated form an intubated patient, displays an extended and resistant biofilm. In previous work, we showed that this peptide did not display anti-biofilm activity at sub-MIC concentrations against the reference strain PAO1 [15]. In this work, we observed similar results for M13513 regarding biofilm inhibition. However, interestingly, the peptide displayed pre-formed biofilm disruption and antimicrobial activity against this strain inside the biofilm. This characteristic highlights the potential of this peptide as a therapeutic candidate against biofilm producing *P. aeruginosa.*

A widely accepted strategy for managing infections associated with drug-resistant bacteria involves the use of combination antimicrobial therapy, particularly with agents that employ distinct mechanisms of action, thus reducing the likelihood of resistance development [32]. Meropenem is a broad-spectrum beta-lactam antibiotic belonging to the carbapenem class. Its mechanism of action involves inhibiting bacterial cell wall synthesis, leading to bacterial cell death. Meropenem targets and binds to PBPs, which are enzymes involved in the final stages of bacterial cell wall synthesis. By inhibiting PBPs, meropenem prevents the cross-linking of peptidoglycan strands, leading to weakened cell walls, which makes the bacteria vulnerable to osmotic pressure, causing cell lysis and ultimately bacterial death. Meropenem is highly resistant to degradation by most beta-lactamases, including extended-spectrum beta-lactamases (ESBLs) and AmpC beta-lactamases, but is hydrolyzed by carbapenemases, like KPC, NDM, OXA, among others. KPC (*Klebsiella pneumoniae* carbapenemase) is a β-lactamase that confers resistance to carbapenems, including meropenem, by breaking down these antibiotics before they can exert their bactericidal effects.

Previous reports showed that polymyxin B, a polypeptide that disrupts the outer cell membrane of Gram-negative bacteria, displays synergy with meropenem [33]. It has been suggested that polymyxin B treatment may enhance intracellular meropenem concentrations (referred to as bioavailability synergy), potentially counteracting meropenem resistance. This resistance arises from enzymatic inactivation by carbapenemases, as mentioned before, decreased cellular entry due to reduced expression of the outer membrane porins, and increased expression of efflux pumps.

A possible explanation of the synergy between meropenem and P6.2, considering that this latter targets the bacterial cellular membrane and makes it more porous, perhaps relays on the presence of efflux pumps and/or mutated porins, a typical characteristic of KPC-producing bacteria. In this way, the porous bacterial membrane allows the entry of more meropenem, counteracting meropenem resistance, as seen for polymyxin B. In this work, synergy between P6.2 and meropenem against a KPC-producing *P. aeruginosa* isolate was evaluated, revealing not only a synergistic interaction but also a noticeable bactericidal activity.

Accordingly, an in vivo assay was conducted in the larvae *G. mellonella*; this infection model has long been established as an adequate study method for bacterial infections [34,35] and particularly for the initial studies of novel anti-pseudomonal treatments [36,37]. These experiments showed that the peptide alone did not display any difference in the survival rate of the infected larvae; however, when P6.2 was administered in combination with a sub optimal concentration of meropenem (1/5 of the effective dose), it showed a significant reduction in larvae death.

## 5. Conclusions

In this study, we investigated the potential in vitro and in vivo synergistic and antibiofilm properties of the designed antimicrobial peptide P6.2. The activity of this peptide was evaluated in carbapenem-resistant *Pseudomonas aeruginosa*, a very important pathogen which is classified as a high-priority group. Although in this work we have used a single isolate and probably additional assays, using a higher number of isolates or other Gram-negative species could be useful to evaluate the impact of the P6.2-meropenem combination. These results, taken together, highlight the potential of the cationic alpha helical AMP P6.2 as a suitable candidate for the development of new treatments in combination with meropenem to fight KPC-harboring *P. aeruginosa* clinical isolates.

## Data Availability

The raw data supporting the conclusions of this article will be made available by the authors on request.

## Supplementary Materials

The following supporting information can be downloaded at: https://www.mdpi.com/article/10.3390/biom15030339/s1, Figure S1. (A) Helical wheel projection diagrams created with HELIQUEST of the peptide, depicting the residues and their relative position in the alpha helix. Yellow circles represent hydrophobic residues and blue circles the positively charged amino acids, uncharged residues are painted in grey (Gly), and negatively charged residues (Glu) in red. The arrow depicts the hydrophobic moment. (B) 3D structure of the peptide P6.2, folded as an alpha helix. The color code for amino acids is the same as for the helical wheel projection. Cartoon created with Chimerax. Table S1. Physicochemical parameters analyzed in silico and experimentally for P6.2. MW: molecular weight (daltons), Ip: isoelectric point, NC: net charge, μH: hydrophobic moment, H: hidrophbicity, and AC: alpha helix content. The percent helix values were determined based on circular dichroism spectra calculated as the mean residue molar ellipticity at 222 nm, in SDS micelles.

## References

[1] Laxminarayan R., Duse A., Wattal C., Zaidi A.K.M., Wertheim H.F.L., Sumpradit N., Vlieghe E., Hara G.L., Gould I.M., Goossens H. (2013). Antibiotic Resistance-the Need for Global Solutions. Lancet Infect. Dis..

[2] Bin Zaman S., Hussain M.A., Nye R., Mehta V., Mamun K.T., Hossain N. (2017). A Review on Antibiotic Resistance: Alarm Bells Are Ringing. Cureus.

[3] Wolfgang M.C., Martin Dozois C., Schmelcher M., Zurich E., Mahlapuu M., Håkansson J., Ringstad L., Björn C. (2016). Antimicrobial Peptides: An Emerging Category of Therapeutic Agents. Front. Cell. Infect. Microbiol. Www.Frontiersin.Org.

[4] Browne K., Chakraborty S., Chen R., Willcox M.D.P., Black D.S., Walsh W.R., Kumar N. (2020). A New Era of Antibiotics: The Clinical Potential of Antimicrobial Peptides. Int. J. Mol. Sci..

[5] Tajer L., Paillart J.C., Dib H., Sabatier J.M., Fajloun Z., Abi Khattar Z. (2024). Molecular Mechanisms of Bacterial Resistance to Antimicrobial Peptides in the Modern Era: An Updated Review. Microorganisms.

[6] Yu G., Baeder D.Y., Regoes R.R., Rolff J. (2018). Predicting Drug Resistance Evolution: Insights from Antimicrobial Peptides and Antibiotics. Proc. R. Soc. B Biol. Sci..

[7] Hollmann A., Martínez M., Noguera M.E., Augusto M.T., Disalvo A., Santos N.C., Semorile L., Maffía P.C. (2016). Role of Amphipathicity and Hydrophobicity in the Balance between Hemolysis and Peptide-Membrane Interactions of Three Related Antimicrobial Peptides. Colloids Surf. B Biointerfaces.

[8] Rima M., Rima M., Fajloun Z., Sabatier J.M., Bechinger B., Naas T. (2021). Antimicrobial Peptides: A Potent Alternative to Antibiotics. Antibiotics.

[9] Wu Y., Jiang S., Li D., Wu Y., Li Q., Wang X., Liu B., Bao H., Wu D., Hu X. (2024). Clinical Efficacy and Safety of Colistin Sulfate in the Treatment of Carbapenem-Resistant Organism Infections in Patients with Hematological Diseases. Infect. Dis. Ther..

[10] Willmann M., Bezdan D., Zapata L., Susak H., Vogel W., Schröppel K., Liese J., Weidenmaier C., Autenrieth I.B., Ossowski S. (2014). Analysis of a Long-Term Outbreak of XDR *Pseudomonas aeruginosa*: A Molecular Epidemiological Study. J. Antimicrob. Chemother..

[11] Bovo F., Amadesi S., Palombo M., Lazzarotto T., Ambretti S., Gaibani P. (2023). Clonal Dissemination of Klebsiella Pneumoniae Resistant to Cefiderocol, Ceftazidime/Avibactam, Meropenem/Vaborbactam and Imipenem/Relebactam Co-Producing KPC and OXA-181 Carbapenemase. JAC Antimicrob. Resist..

[12] WHO Bacterial Priority Pathogens List (2024). 2024: Bacterial Pathogens of Public Health Importance to Guide Research, Development and Strategies to Prevent and Control Antimicrobial Resistance.

[13] Tenover F.C., Nicolau D.P., Gill C.M. (2022). Carbapenemase-Producing *Pseudomonas aeruginosa*—An Emerging Challenge. Emerg. Microbes Infect..

[14] Maturana P., Martinez M., Noguera M.E., Santos N.C., Disalvo E.A., Semorile L., Maffia P.C., Hollmann A. (2017). Lipid Selectivity in Novel Antimicrobial Peptides: Implication on Antimicrobial and Hemolytic Activity. Colloids Surf. B Biointerfaces.

[15] Martínez M., Polizzotto A., Flores N., Semorile L., Maffía P.C. (2020). Antibacterial, Anti-Biofilm and in Vivo Activities of the Antimicrobial Peptides P5 and P6.2. Microb. Pathog..

[16] Pasteran F., Faccone D., Gomez S., De Bunder S., Spinelli F., Rapoport M., Petroni A., Galas M., Corso A. (2012). Detection of an International Multiresistant Clone Belonging to Sequence Type 654 Involved in the Dissemination of KPC-Producing *Pseudomonas aeruginosa* in Argentina. J. Antimicrob. Chemother..

[17] CLSI (1999). Methods for Determining Bactericidal Activity of Antimicrobial Agents. Approved Guideline.

[18] White R.L., Burgess D.S., Manduru M., Bosso J.A. (1996). Comparison of Three Different in Vitro Methods of Detecting Synergy: Time-Kill, Checkerboard, and E Test. Antimicrob. Agents Chemother..

[19] Faccone D., Veliz O., Corso A., Noguera M., Martínez M., Payes C., Semorile L., Maffía P.C. (2014). Antimicrobial Activity of de Novo Designed Cationic Peptides against Multi-Resistant Clinical Isolates. Eur. J. Med. Chem..

[20] Sánchez-Gómez S., Lamata M., Leiva J., Blondelle S.E., Jerala R., Andrä J., Brandenburg K., Lohner K., Moriyón I., Martínez-De-Tejada G. (2008). Comparative Analysis of Selected Methods for the Assessment of Antimicrobial and Membrane-Permeabilizing Activity: A Case Study for Lactoferricin Derived Peptides. BMC Microbiol..

[21] Cappelletty D.M., Rybak M.J. (1996). Comparison of Methodologies for Synergism Testing of Drug Combinations against Resistant Strains of *Pseudomonas aeruginosa*. Antimicrob. Agents Chemother..

[22] Barry A.L., Craig W.A., Nadler H., Reller L.B., Sanders C.C., Swenson J.M. (2016). M26-A: Methods for Determining Bactericidal Activity of Antimicrobial Agents; Approved Guideline. Int. Clin. Lab. Stand. Guidel. ICLS.

[23] Nickerson R., Thornton C.S., Johnston B., Lee A.H.Y., Cheng Z. (2024). *Pseudomonas aeruginosa* in Chronic Lung Disease: Untangling the Dysregulated Host Immune Response. Front. Immunol..

[24] Abraham P., George S., Kumar K.S. (2014). Novel Antibacterial Peptides from the Skin Secretion of the Indian Bicoloured Frog Clinotarsus Curtipes. Biochimie.

[25] Akbari R., Hakemi Vala M., Sabatier J.M., Pooshang Bagheri K. (2022). Fast Killing Kinetics, Significant Therapeutic Index, and High Stability of Melittin-Derived Antimicrobial Peptide. Amino Acids.

[26] Lofton H., Pränting M., Thulin E., Andersson D.I. (2013). Mechanisms and Fitness Costs of Resistance to Antimicrobial Peptides LL-37, CNY100HL and Wheat Germ Histones. PLoS ONE.

[27] Xu S., Tan P., Tang Q., Wang T., Ding Y., Fu H., Zhang Y., Zhou C., Song M., Tang Q. (2023). Enhancing the Stability of Antimicrobial Peptides: From Design Strategies to Applications. Chem. Eng. J..

[28] Chen S.P., Chen E.H.L., Yang S.Y., Kuo P.S., Jan H.M., Yang T.C., Hsieh M.Y., Lee K.T., Lin C.H., Chen R.P.Y. (2021). A Systematic Study of the Stability, Safety, and Efficacy of the de Novo Designed Antimicrobial Peptide PepD2 and Its Modified Derivatives Against Acinetobacter Baumannii. Front. Microbiol..

[29] Zhao A., Sun J., Liu Y. (2023). Understanding Bacterial Biofilms: From Definition to Treatment Strategies. Front. Cell. Infect. Microbiol..

[30] Hanot M., Lohou E., Sonnet P. (2025). Anti-Biofilm Agents to Overcome *Pseudomonas aeruginosa* Antibiotic Resistance. Pharmaceuticals.

[31] de Pontes J.T.C., Borges A.B.T., Roque-Borda C.A., Pavan F.R. (2022). Antimicrobial Peptides as an Alternative for the Eradication of Bacterial Biofilms of Multi-Drug Resistant Bacteria. Pharmaceutics.

[32] Ardebili A., Izanloo A., Rastegar M. (2023). Polymyxin Combination Therapy for Multidrug-Resistant, Extensively-Drug Resistant, and Difficult-to-Treat Drug-Resistant Gram-Negative Infections: Is It Superior to Polymyxin Monotherapy?. Expert. Rev. Anti Infect. Ther..

[33] Wickremasinghe H., Yu H.H., Azad M.A.K., Zhao J., Bergen P.J., Velkov T., Zhou Q.T., Zhu Y., Li J. (2021). Clinically Relevant Concentrations of Polymyxin B and Meropenem Synergistically Kill Multidrug-Resistant *Pseudomonas aeruginosa* and Minimize Biofilm Formation. Antibiotics.

[34] Barton T.E., Duignan L., Kadioglu A., Fothergill J.L., Neill D.R. (2024). Galleria Mellonella as an Antimicrobial Screening Model. JoVE.

[35] Ménard G., Rouillon A., Cattoir V., Donnio P.Y. (2021). Galleria Mellonella as a Suitable Model of Bacterial Infection: Past, Present and Future. Front. Cell Infect. Microbiol..

[36] Tsai C.J.Y., Loh J.M.S., Proft T. (2016). Galleria Mellonella Infection Models for the Study of Bacterial Diseases and for Antimicrobial Drug Testing. Virulence.

[37] Hill L., Veli N., Coote P.J. (2014). Evaluation of Galleria Mellonella Larvae for Measuring the Efficacy and Pharmacokinetics of Antibiotic Therapies against *Pseudomonas aeruginosa* Infection. Int. J. Antimicrob. Agents.

