# Supplementary material for: In Vitro and In Vivo Evaluation of the De Novo Designed Antimicrobial Peptide P6.2 Against a KPC-Producing P. aeruginosa Clinical Isolate
Source: Biomolecules. 2025 Feb 27;15(3):339. doi: 10.3390/biom15030339 (PMC11940343; doi:10.3390/biom15030339)
Supplement: Supplementary file 1 [file biomolecules-15-00339-s001.zip › biomolecules-3471037-supplementary.pdf]

Supplementary material

Suplementary Figure S1

Sequence (C term amidated): *GLLRKWGKKWKEFLRRVWK*

A)

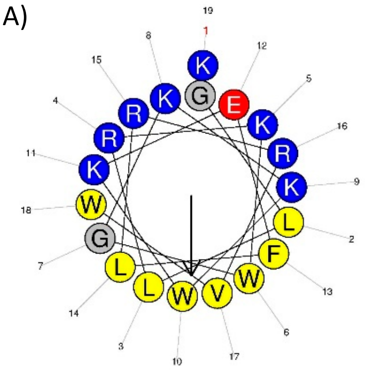

B)

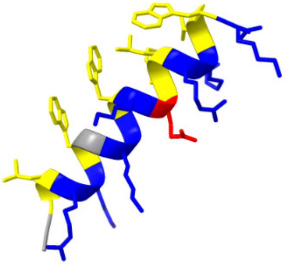

A) Helical wheel projection diagrams created with HELIQUEST of the peptide, depicting the residues and their relative position in the alpha helix. Yellow circles represent hydrophobic residues and blue circles the positively charged aminoacids, uncharged residues are painted in grey (Gly), and negatively charged residues (Glu) in red. The arrow depicts the hydrophobicmoment.

B) 3D structure of the peptide P6.2, folded as an alpha helix. The color code for amino acids is the same as for the helical wheel projection. Cartoon created with Chimerax

Table S1. Physicochemical parameters analyzed in silico and experimentally for P6.2. MW: molecular weigth (daltons), Ip: isoelectric point, NC: net charge, μH: hydrophobic moment, H: hidrophbicity, AC: alpha helix content. The percent helix values were determined based on circular dichroism spectra calculated as the mean residue molar ellipticity at 222 nm, in SDS micelles. *a: http://web.expasy.org/compute_pi/. **b: http://heliquest.ipmc.cnrs.fr/cgi-bin/ComputParamsV2.py.

**Physichochemical parameters**	**MW[a]**	**IP[a]**	**NC[b]**	**μH[b]**	**H[b]**	**%AH**
P6.2	2515.09	11.75	7	0.793	0.328	46
